# Genomic and Metagenomic Analysis of Diversity-Generating Retroelements Associated with *Treponema denticola*

**DOI:** 10.3389/fmicb.2016.00852

**Published:** 2016-06-03

**Authors:** Sutichot Nimkulrat, Heewook Lee, Thomas G. Doak, Yuzhen Ye

**Affiliations:** ^1^School of Informatics and Computing, Indiana University, BloomingtonIN, USA; ^2^Computational Biology Department, School of Computer Science, Carnegie Mellon University, PittsburghPA, USA; ^3^Department of Biology, Indiana University, BloomingtonIN, USA; ^4^National Center for Genome Analysis Support, Indiana University, BloomingtonIN, USA

**Keywords:** diversity-generating retroelements, *Treponema denticola*, human microbiome, template region, reverse transcriptase

## Abstract

Diversity-generating retroelements (DGRs) are genetic cassettes that can produce massive protein sequence variation in prokaryotes. Presumably DGRs confer selective advantages to their hosts (bacteria or viruses) by generating variants of target genes—typically resulting in target proteins with altered ligand-binding specificity—through a specialized error-prone reverse transcription process. The only extensively studied DGR system is from the *Bordetella* phage BPP-1, although DGRs are predicted to exist in other species. Using bioinformatics analysis, we discovered that the DGR system associated with the *Treponema denticola* species (a human oral-associated periopathogen) is dynamic (with gains/losses of the system found in the isolates) and diverse (with multiple types found in isolated genomes and the human microbiota). The *T. denticola* DGR is found in only nine of the 17 sequenced *T. denticola* strains. Analysis of the DGR-associated template regions and reverse transcriptase gene sequences revealed two types of DGR systems in *T. denticola*: the ATCC35405-type shared by seven isolates including ATCC35405; and the SP32-type shared by two isolates (SP32 and SP33), suggesting multiple DGR acquisitions. We detected additional variants of the *T. denticola* DGR systems in the human microbiomes, and found that the SP32-type DGR is more abundant than the ATCC35405-type in the healthy human oral microbiome, although the latter is found in more sequenced isolates. This is the first comprehensive study to characterize the DGRs associated with *T. denticola* in individual genomes as well as human microbiomes, demonstrating the importance of utilizing both individual genomes and metagenomes for characterizing the elements, and for analyzing their diversity and distribution in human populations.

## Introduction

**FIGURE 1:**
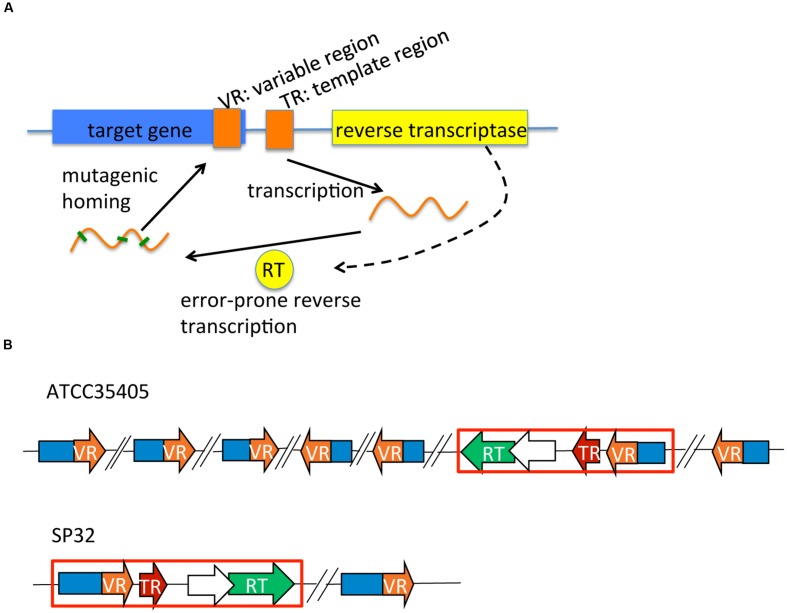
**DGR systems: a schematic representation of a generic DGR system, and those identified in *Treponema denticola* isolates.** **(A)** A typical DGR system consists of a RT gene, a TR, and a VR located in a target gene. **(B)** DGR systems in *T. denticola* isolates vary in terms of the type (related to the RT and TR) and the number of target genes. Only two selected systems (representing the two types) are shown here. The red boxes highlight the essential (core) modules of each DGR system containing the RT (in green), the TR (in red), and one target gene containing the VR (in orange). Predicted accessory genes (which overlap with the RT genes) are shown as open arrows. Target genes containing VRs that are far away from the core modules are also shown in the figure—with back-slashes used to indicate substantial distances between these remote target genes and the core module locus. See our tDGR website for the coordinates and names of the target genes found in all *T. denticola* isolates.

Diversity-generating retroelements were first observed in the *Bordetella* bacteriophage BPP-1, which generates genetic variations at the 3′ end of the *mtd* (major tropism determinant) gene. The *mtd* gene encodes a C-type lectin fold that binds a *Bordetella* outer membrane receptor protein called pertactin (Leininger et al., 1991; Cotter and Miller, 1997; Liu et al., 2002). The *mtd* gene is target-diversified by a reverse transcriptase (RT, whose gene is in the neighborhood of the *mtd* gene), guided by a template region (TR), producing target protein variants with altered ligand-binding specificity (Liu et al., 2002; Miller et al., 2008; Dai et al., 2010) (see **Figure 1A** for a schematic representation of DGR systems). First, the TR is transcribed and its RNA serves as the fundamental genetic pattern for RT to synthesize variable cDNA (Liu et al., 2002; Guo et al., 2008). The variable cDNA, which has been hyper-mutagenized at adenine positions of the TR sequence, is copied into the 3′-end variable region (VR) of the *mtd* gene (the homing process). In each round, the last mutant VR form is replaced with a new derivative from the “archival” TR. This process generates highly diversified protein variants of the target gene, and allows the phage to utilize various receptors on the host.

Diversity-generating retroelement systems are known to produce massive protein sequence variation in prokaryotes—equivalent to mammalian immunoglobins—and are one of the main sources of extreme interpersonal diversity of human gut viruses (Minot et al., 2012). DGR target proteins are known to form a C-type lectin (CLec) fold (Le Coq and Ghosh, 2011), a structural framework that can tolerate massive variations. Arambula et al. (2013) characterized a DGR in *Legionella pneumophila*—within a horizontally acquired genomic island—and identified related DGRs in clinical *L. pneumophila* isolates that encode unique target surface-displayed C-type lectin proteins with homologous VRs, demonstrating the adaptability of DGR components. In addition to the C-type lectin (CLec) fold, the other instances of protein folds that tolerate massive sequence variation for the sake of increasing binding diversity, include the immunoglobulin fold (the most extensively characterized instance) and the leucine-rich (LRR) repeat (which is found in variable lymphocyte receptors of jawless vertebrates, a component of their adaptive immune system) (Herrin and Cooper, 2010). We note that pathogenic organisms have developed different mechanisms for producing diverse surface proteins and structures, including antigenic variation arisen by either mutation or recombination, important their survival within hosts and transmission between hosts (Brunham et al., 1993; van der Woude and Baumler, 2004; Coutte et al., 2009). However, compared to other mechanisms, the DGR systems can potentially generate a much greater scale of sequence diversity localized in a target gene (Schillinger et al., 2012).

In our study, we used *Treponema denticola* to investigate DGR biology. *T. denticola* is a spirochete bacterium that occupies the human mouth and is associated with periodontal disease. Computational predictions (Medhekar and Miller, 2007) and structural evidence for the target gene (Le Coq and Ghosh, 2011) suggest that adenine mutagenesis of DGRs is conserved in *T. denticola*, and its DGR contains a core module consisting of VR, TR, and RT, as seen for DGRs in other organisms (Liu et al., 2002; Schillinger et al., 2012). The targeted gene of hyper-variability in *T. denticola* encodes the C-lectin fold-containing protein *Treponema* variable protein A (TvpA), instead of Mtd as in *Bordetella* (Le Coq and Ghosh, 2011). It was estimated that the DGR system in this species could produce ∼6 × 10^20^ TvpA variants. The function of TvpA has not been identified, but it has been found to form a structure similar to Mtd (although with low sequential similarity). In addition, TvpA and other *T. denticola* DGR variable proteins have predicted lipoprotein signal sequences that likely target these proteins to the outer surface of the spirochete, suggesting a role of *T. denticola* DGR variable proteins in mediating interactions with other organisms (Schulze and Zuckert, 2006; Le Coq and Ghosh, 2011), including their human host. Periodontitis requires a progressive set of complex interactions, from multi-species bacterial biofilms, to bacterial attachment to host cells, and avoidance of host immunity (Dashper et al., 2011; Fenno, 2012; Hajishengallis, 2015). While the targets of the *Treponema* TvpA are not yet known, the hypervariable C-type lectin fold proteins of *T. denticola* are an intriguing topic for future examination, for their involvement in periodontal disease progression.

To better understand the natural functioning of the *T. denticola* DGR system, we combined an analysis of genomic data with metagenomic data sets. The genomic dataset is a collection of 17 complete and draft *T. denticola* genomes; the metagenomic datasets come from the NIH Human Microbiome Project (HMP), consisting of ∼100 bp metagenomic reads of >700 samples from multiple body sites (including oral cavity, nasal cavity, skin, gastrointestinal tract, and urogenital tract) of 242 healthy adults (all included subjects were between the ages of 18 and 40 years and had passed a screening for systemic health based on oral, cutaneous and body mass exclusion criteria) (HMP, 2012). These two datasets complement each other: complete/draft genomes provide the essential genomic context for studying the DGR systems, while the HMP datasets allow us to study the (unbiased) distribution of the *T. denticola* associated DGR systems in the human population, and the natural diversity generated by these DGR systems.

Despite the DGR research in *Bordetella* species, expansion of the investigation to additional organisms will extend our knowledge of DGR systems. The first studies of DGR in the human microbiome (Minot et al., 2012) and in a pathogen associated with human disease (Arambula et al., 2013) were published. Recently DGR systems have been discovered in archaea and archaeal viruses, including ANMV-1, an archaeal phage, and two operational taxonomic units of uncultivated subterranean *Nanoarchaeota* (Paul et al., 2015). Our previous study (Ye, 2014) revealed a great diversity of DGR systems in the human microbiome, using metagenomic assemblies. However, despite the large number of DGR systems identified from the human microbiome, none of the identified RTs share more than 40% sequence identity with the *T. denticola* DGR RT, probably because of *T. denticola’s* low abundance. Taking advantage of the unique DGR template sequence (TR) associated with *T. denticola*, in this study we searched for potential *T. denticola* associated DGR systems, using the TR as the tag. We found that *T. denticola*-associated DGR systems are found frequently in the human population. Using the large number of reads derived from the template and variable sequences of the DGR systems, we were able to study the distribution of *T. denticola*-associated DGR systems in the human population, and study the mutation patterns in the VRs. We found that *T. denticola* contains multiple TR variants, which generate a different spectrum of amino acid variation in *T. denticola* TR and VR, in comparison to other DGR systems. The genomic and metagenomic data shows that there are many variant TRs, and that DGR systems of *T. denticola* in humans are prevalent and diverse in the human population, with the most abundant type being the SP32-type TR variant.

## Materials and Methods

### Identification of DGR in *T. denticola* Genomes

Genome sequences for 17 strains of *T. denticola* were downloaded from the NCBI website and the Broad Institute website [Human Microbiome U54 initiative, Broad Institute (broadinstitute.org)], and analyzed to determine the orientation of the core DGR system. Among the strains, ATCC35405 is one of the routinely used laboratory strains; it was initially isolated and designated as the type strain by Chan et al. (1993), and was the first *T. denticola* strain to have its genome sequenced (Seshadri et al., 2004).

We used DGRscan (Ye, 2014) to determine if these genomes carry DGR systems, and if so, the location and sequence of the TR, VR, and RT. An isolate is considered to carry a DGR locus if it contains at least a TR, a gene encoding RT, and one variable-region-containing target gene (but a species may contain multiple variable genes). DGRscan is a computational tool we developed for either *de novo* identification of DGR systems (based on prediction of potential template-VR pairs, using or without using prediction of putative RT genes to constrain the search), or similarity-based searches of DGR systems using known TRs as the reference (to identify TR-VR pairs that share similarity with reference TRs). Here we used DGRscan (the *de novo* search mode) to search for putative DGR systems in *T. denticola* genomes, using similarity search results of RT genes to constrain the identification of DGR systems. Prediction of RT genes was done by BLAST against 155 reference RT genes collected based on a previous study (Schillinger et al., 2012), which supported that DGR RT genes form a distinct and well-defined clade in the tree of RTs (Doulatov et al., 2004). For genomes lacking DGR systems, we further confirmed the results using *de novo* predictions by DGRscan without applying any constraints. To extract target genes containing the VRs, we first blasted predicted *T. denticola* genes against identified TRs. Genes with regions similar to TRs (with a minimum alignment length of 30) were considered putative target genes, and were further checked for hyper-mutations involving changes of adenines to other bases, assisted by DGRscan. We note that this approach allows us to identify target genes that contain VRs similar to identified TRs, which otherwise can be very diverse.

### Tree of *T. denticola*

To understand the dynamics of the DGR systems in the *T. denticola* linage, we collected 31 phylogenetic marker genes (Ciccarelli et al., 2006) for the *Treponema* genomes and built a phylogenetic tree—note that all the isolates contained all 31 marker genes. It was shown that these marker genes enable automatic reconstruction of a highly resolved tree of life (Ciccarelli et al., 2006), and this approach has been widely adopted for estimating species trees (Rinke et al., 2013). We aligned the amino acid sequences of the marker genes using MUSCLE (Edgar, 2004)—separately for each gene—and used Gblocks to exclude poorly aligned regions (Castresana, 2000; Talavera and Castresana, 2007). The aligned amino acid sequences were then concatenated and imported to MEGA (Tamura et al., 2013) for tree construction, with the JTT (Jones–Taylor–Thornton) model. The bootstrap number was 500. To position the root of the tree, we included organisms phylogenetically distant from *T. denticola* including *Spirochaeta coccoides*, *Shewanella denitrificans*, and *Vibrio cholera*. This rooting oriented the *T. denticola* branches, and allowed us to identify multiple *T. denticola* clades that have seemingly independent acquisitions of DGR systems.

### DGR in the Human Microbiome

The HMP datasets were analyzed to identify *T. denticola* DGR reads among different human populations and body positions. Considering that the human population may contain *T. denticola*-associated DGR variants different from the ones we identified in genome assemblies, we deployed an iterative procedure: identification of potential VR/TR using known TRs as a reference, assembly of identified reads (hoping to assemble different TRs), and then identification of potential VR/TR reads using known TRs and newly identified TRs as the reference again.

For the initial identification, we used 3 TR variants identified from sequenced isolates (ATCC35405, SP32, and SP37) as the reference to search against the HMP dataset. These three TR sequences are sufficient to cover the observed diversity of the TRs in the sequenced isolates: SP32-TR and ATCC35405-TR share ∼81% sequence identity, and SP37-TR and ATCC35405-TR only differ at one position. We note that it is important to use unassembled sequencing reads (instead of assemblies) for searches, as *T. denticola* species are rare in healthy human population and therefore poorly represented in assemblies of the HMP datasets. We only kept read hits that were at least 50 bp long and with 70% sequence identity to one of the reference TRs. Each of those candidate reads was aligned to the most similar TR reference, then categorized as a TR or VR, using a custom Python script of the Smith-Waterman local alignment that tolerates sequence mutations of DGR [implemented in DGRscan (Ye, 2014)]. HMP reads that were considered as TR had to align with one of the reference TRs with only two mismatches. On the other hand, HMP reads that were recognized as VR had to contain at least five mutations and 50% of those mutations had to be adenine to another nucleotide when aligned to a TR.

Predicted TR reads were employed by the SOAPdenovo assembler to identify additional (and hopefully complete) TRs in the HMP datasets (Luo et al., 2012). Newly assembled TRs from SOAPdenovo were combined with the list of original reference TRs reiterating in BLASTn searches against the HMP dataset. This reiteration was done to increase the pool of potential TR/VR reads for analysis. For the purpose of this study, the repetition of BLASTn was done only once, but it could be repeated multiple times.

### Analyzing the Hypervariation Patterns of the DGR Systems

Since we detected multiple TR variants in the HMP datasets, we needed to eliminate biases that would obscure predicted mutagenesis from TRs to VRs: we need to identify the variations found between TRs and exclude them from study of the spectrum of nucleotide and amino acid substitutions in the DGR systems involving TR to VR mutations. Specifically, we prepared a multiple alignment of putative TR sequences (predicted by DGRscan), and used only the positions that are conserved among the TR sequences for studies of TR to VR variation patterns. The conserved positions are those where no more than two reads differ from the rest of the column; columns of residues in the TR multiple alignment that had more than two differences were considered as non-conserved positions.

The protein structure of TvpA was collected from Protein Data Bank (accession no. 2Y3C), and three-dimensional images were built using PyMol^1^. The VR of TvpA is located at the C-terminus of the protein, from residue 284 to 329 (Le Coq and Ghosh, 2011), but in our analysis, we only used residues 286 to 328. We used TvpA to map the preferential positions and hotspots of mutagenesis of the *T. denticola* DGR system at the amino acid level. The variable residues were acquired from analysis of DGR hypervariation patterns, and then mapped to TvpA according to the multiple alignment of reference TRs.

## Results

### Features of DGR in *T. denticola*

We identified complete DGR systems in 9 of the 17 assembled *T. denticola* genomes, each having at least one VR, TR, and RT (the core DGR component; **Table 1**; **Figure 1B**); and additional target genes (containing VRs), separate from the core modules. These extra target genes may be leftover components from previous DGR systems or duplications of the core modules, but may still be functional, since one copy of RT and the TR sequence will be sufficient in a genome. In addition, these extra target genes contain VRs that are likely to be hypervariated from the same TR. The different *T. denticola* isolates each contain between two to seven target genes; and some also contain additional “suspicious” target genes (either the genes are truncated or contain excessive mutations in the VR). For example, there are seven target genes [which were reported by Le Coq and Ghosh (Le Coq and Ghosh, 2011)] that contain VRs in the ATCC35405 isolate: the TR is between 2,304,822 and 2,304,692 bp, and the seven target genes are located at 603,634-604,794 (gene ID: TDE0572), 967,268-968,380 (TDE0945), 1,085,350-1,085,955 (TDE1056), 2,126,709-2,125,639 (reverse strand, TDE2101), 2,277,636-2,276,644 (reverse strand, TDE2239), 2,306,010-2,305,021 (reverse strand, TDE2269), and 2,542,147-2,541,062 (reverse strand, TDE2515). Although the seven VRs are all different from each other (sharing about 77% sequence identity), most of their deviations from the TR are at positions in the template where there is an adenine (56 columns in the multiple alignment of the TR and the seven VRs involve mutations, and among them 38 have adenine in the TR; see **Supplementary Figure S1** for the multiple alignment).

**Table 1:** *T. denticola* isolates that contain components of DGR system.

Isolates	# of TR	# of RT	# of target genes	# of suspicious target gene
**ATCC35405**	**1**	**1**	**7**	
**ATCC33521**	**1**	**1**	**7**	
**ATCC35404**	**1**	**1**	**7**	
**OTK**	**1**	**1**	**4**	**1 (excessive mutations)**
**SP32**	**1**	**1**	**2**	**1 (truncated, 91 aa)**
**SP33**	**1**	**1**	**2**	**1 (truncated, 92 aa)**
**SP23**	**1**	**1**	**7**	**1 (excessive mutations)**
**SP37**	**1**	**1**	**7**	**1 (excessive mutations)**
**SP44**	**1**	**1**	**7**	**1 (excessive mutations)**
ATCC33520	0	0	4	
AL-2	0	0	6	
ASLM	0	0	5	1 (truncated, 136 aa)
F0402	0	0	3	1 (truncated, 58 aa)
H-22	0	0	4	
H1-T	0	0	2	1 (excessive mutations)
MYR-T	0	0	2	1 (excessive mutations)
US-Trep	0	0	5	1 (truncated, 136 aa)


*Complete DGR systems are shown in bold. Target genes with truncated genes (encoding <140 aa) or containing excessive mutations in the VR are considered to be suspicious.*

We utilized PHMMER (Finn et al., 2011) to predict the function of genes subjected to hypermutagenesis in *T. denticola*. All of the genes from 9 isolates were predicted as FGE-sulfatase (Sardiello et al., 2005). So all the identified target genes had C-type lectin fold domains similar to that of *Bordetella*, since the FGE domain forms a C-type lectin fold; thus, all genes targeted by the *T. denticola* DGR system are similar to previously reported TvpA. Comparison of the VR sequences in the target genes to the corresponding TR sequences revealed that 22 out of 43 amino acids are unmodified in the VR sequences. Although the majority of hypervariations are asparagine codons to other residues’ codons, we also observed hypervariations involving lysine codons, which we also observed in the HMP datasets (see below). In addition, most of the target genes we identified (62%, including those from *T. denticola* strains that lack core DGR systems) encode proteins that contain lipoprotein signal peptides, according to PRED-LIPO prediction (Bagos et al., 2008), which is consistent with previous studies (Juncker et al., 2003; Schulze and Zuckert, 2006; Le Coq and Ghosh, 2011). For example, lipoprotein signal peptides were predicted with strong evidence in seven out of eight target proteins found in SP23; the only exception is HMPREF9731_01298, which was predicted to be a secreted protein by PRED-LIPO. Among seven target proteins in ATCC35405, three were predicted to be lipoproteins, including TDE0572, TDE2101, and TDE2269. Except for SP32, which has two target proteins neither predicted to be a lipoprotein, all other isolates each have at least two target lipoproteins. See **Supplementary Figure S2** for selected examples of lipoprotein signal peptide prediction and predicted lipoboxes in SP23 and ATCC35405.

For all nine DGR isolates, there is an accessory gene between the TR and RT (shown as open arrows in **Figure 1B**). Functional annotation of these accessory genes suggested they encode proteins containing a Helicase or the RNaseD C-terminal domain (HRDC). HRDC is the domain of RecQ helicases that denatures DNA duplexes and mediates interactions between DNA and other proteins (Wu et al., 2005). The existence of the accessory gene in every DGR and compact clustering of this gene and other core modules strongly suggest that HRDC assists RT during adenine hyper-mutagenesis. Interestingly, the accessory genes overlap with the neighboring RT genes, in all nine DGR-containing *T. denticola* isolates: for example, the putative RT gene in the ATCC35404 isolate is located between 2,303,132 and 2,304,118 bp (reverse strand), and its putative accessory gene is located between 2,304,072 and 2,304,554 bp (reverse strand), indicating an overlap of 47 bps, in different frames.

We also checked if there are regions similar to IMH (Initiation of Mutagenic Homing) sites in the 3′-ends of the VRs in *T. denticola*. It was found in *Bordetella phage* that the 21 bp IMH site differs from its counterpart at the 3′ end of TR, IMH^∗^, by only five bp, and this difference prevents TR from being a homing recipient (Guo et al., 2008). Identical 14 bp G/C-only sequences [(G/C)_14_] are found in the sequence immediately upstream of IMH and IMH^∗^. We observed almost identical 3′-ends in *T. denticola’s* VR and TR, with a few different positions—they are likely the IMH and IMH^∗^ sites in the *T. denticola*’s TR and VR. Similar to *Bordetella* phage, *T. denticola*’s VR and TR contain identical sequences of about 14 bps long (14 bps in ATCC35405, and 13 bps in SP32) immediately upstream of predicted IMH and IMH^∗^, but the sequences are not G/C-only (see **Supplementary Figure S3**).

### Diversity of the DGR Systems in *T. denticola* Isolates

**FIGURE 2:**
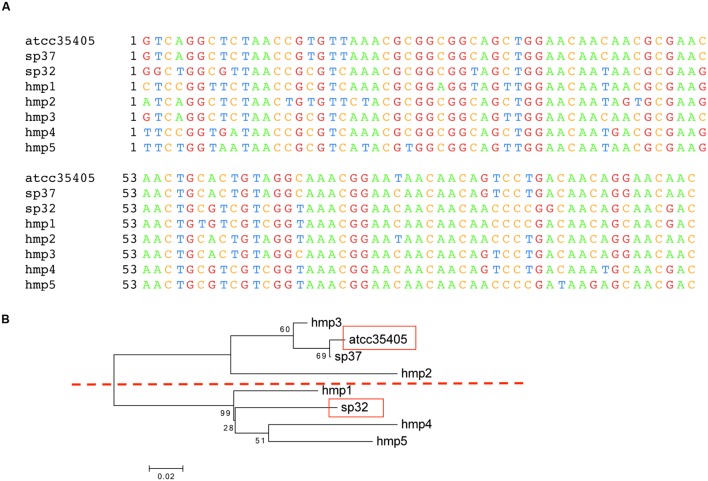
**Template region variants in isolates and the HMP dataset.** **(A)** Multiple sequence alignment of the three TR variants found in sequenced isolates (ATCC35405, SP37, and SP32) and five TR variants identified from HMP datasets (HMP1–5). The alignment is truncated, not showing the 3′-ends, as some of the TR variants identified from the HMP datasets are incomplete (see **Supplementary Figure S4** for the alignment of full length TR sequences identified from the isolates). **(B)** A neighbor-joining tree of the TRs, in which the ATCC35405 and SP32 branches are highlighted in red boxes, computed using MEGA (Tamura et al., 2013).

**FIGURE 3:**
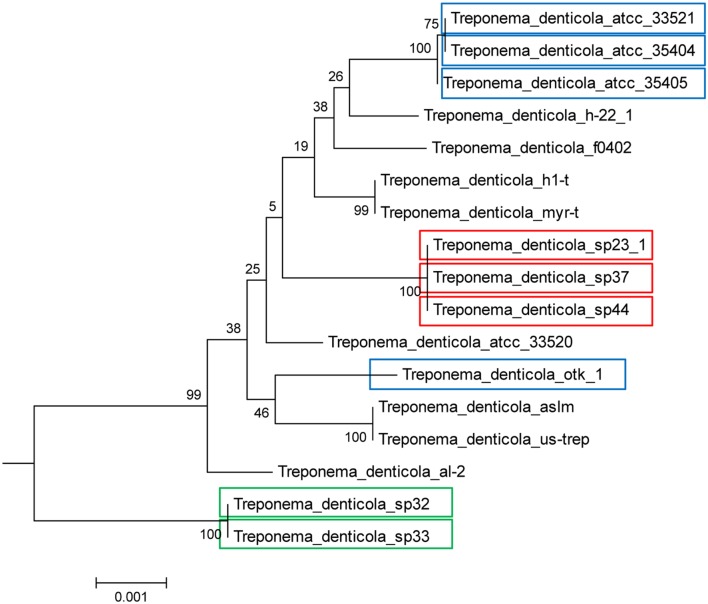
**Presence of DGR systems in the *T. denticola* lineage, established with 31 phylogenetic marker genes.** Isolates with colored boxes indicates those isolates containing full DGR systems, with a TR, VR, and RT. Different colors illustrate the different types of TR contained in each isolate. The root of the tree is established with other *Treponema* species and more distantly related bacteria. Blue represents the ATCC35405-type TR, red represents the SP37-type TR, and green represents the SP32-type TR. Note that the ATCC35405-type TR (blue) and the SP37-type TR (red) only differ at one position, while the SP32-type TR (green) is distinct, with a 19% difference in nucleotide sequence as compared to the ATCC35405-type. See Methods for the construction of the tree.

We find two main types of DGR loci among the nine *T. denticola* strains with identified DGR systems. The majority of these isolates shared the same TR as ATCC35405 TR (the ATCC35405-type); SP37, SP23-1, and SP44 share a variant type (the SP37-type), containing a TR that differs from the ATCC35405-type TR at one position (from thymine to cytosine at position 75, a silent site). SP32 and SP33 contain a TR (the SP32-type TR) that is distinct from the other isolates, sharing only 81% sequence identity with ATCC35405-type TR. **Figure 2A** shows the alignment between the ATCC35405- and SP32-type TRs (and the TRs identified from the HMP datasets). The evolutionary tree of the *T. denticola* genus suggests that isolates with similar types of TR are more closely related to one another than ones with different TRs, and generates three clades of TR variants (**Figure 3**); only the OTK strain is placed outside the ATCC35405 clade, although it has the ATCC35405 type TR. Overall, multiple gains, and possibly multiple losses, of DGR systems from the *T. denticola* species seem necessary to explain this distribution; there are some short branches with weak support (low bootstrap values) in the tree, but other proposed gains are robustly supported.

**FIGURE 4:**
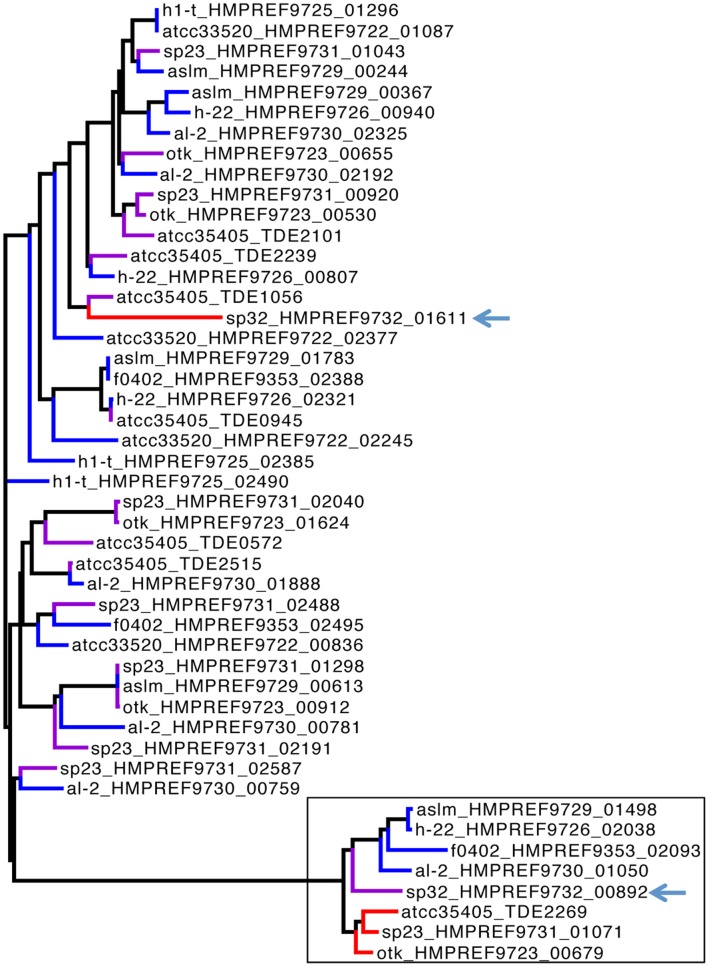
**Phylogenetic tree of target genes.** The tree was built by FastTree (Price et al., 2009), using a multiple alignment of the genes at the amino level by MUSCLE (Edgar, 2004). Only genes from ten representative isolates are presented here for clarity—some isolates have very similar genomes (e.g., ATCC35405, ATCC35404, and ATCC33521 are very similar so only ATCC35405 was used for the tree). We denote the genes by isolate names followed by gene IDs in the tree. The red, violet, and blue branches highlight the target genes that are part of the core DGR components, target genes that are remote to the core DGR components, and target genes found in the isolates that lack the core DGR components (without RT genes and TRs), respectively. The black box highlights the branch of “core” target genes, containing mainly the target genes found in core components of the DGR systems (red) and the target genes that we believe are remnants of the core DGR components in the species lacking complete DR systems (blue). The only exception is SP32 (whose target genes are highlighted with arrows in the figure): the remote target gene (HMPREF9732_00892) shares more sequence similarity with the core target genes in other species (red or blue) than its core target gene (HMPREF9732_01611).

The RTs of isolates (SP32 and SP33) containing the SP32-type TR shared ∼70% nucleotide sequence identity with the RTs of the other isolates. This difference is consistent with their distinct TR sequence, and consistent with the host genomes basal position in the phylogeny, indicating that SP32 and SP33 were evolutionarily quite different from the other DGR-containing isolates. Further analysis of HRDC proteins revealed two groups of HRDC proteins: SP32 and SP33 share identical HRDC proteins, which is distinct from the HRDC proteins shared by the other isolates with complete DGR components. Thus, we have identified two distinct types of DGR systems in available *T. denticola* genomes. Similarly, the target gene (HMPREF9732_01611), found in the core DGR component in isolate SP32, is also distinct from the core target genes found in the isolates that contain the ATCC35405-type DGR systems, as shown in **Figure 4** (target genes in the core DGR components are highlighted in red in the tree).

Interestingly, isolate SP32 has two target genes, and the remote target gene (HMPREF9732_00892)—which is distant from the core DGR component—is actually more similar to core target genes found in the ATCC35405-type DGR systems. In addition, this gene contains a VR that is more similar to the ATCC35405-type TR than the SP32-type TR: comparing to the ATCC35405-type TR, the VR has 21 mutations, 19 of which involve adenines in the TR. These results indicate that the remote gene in SP32 is likely a leftover of the ATCC35405-type DGR component that was in SP32 (an alternative explanation would be that this target gene was acquired alone). This also supports our hypothesis of multiple gains/losses of the DGR systems in *T. denticola* species: the SP32 isolate seems to have gained a complete DGR system (the SP32-type), with a partial loss of the ATCC35405-type DGR system. We note that we have also identified “target genes” in *T. denticola* isolates that lack core DGR systems (see **Table 1**; **Figure 4**). Neither RT genes nor TRs were identified in these isolates; nevertheless, the “target genes” found in these isolates contain VRs that differ from TR sequences (identified in DGR-containing *T. denticola* isolates) mostly at positions corresponding to adenines in the TRs. These target genes are likely leftovers from losses of the core DGR systems. For example, six target genes were identified in *T. denticola* al-2; one of the target genes (HMPREF9730_01050) is very similar to the target genes in the core ATCC35405-type DGR systems (as shown in **Figure 4**), indicating an incomplete loss of the core DGR system in this isolate, and the other five are more similar to the target genes that are distant to the core DGR systems.

### DGR Systems in Human Microbiomes

Using the three TR sequences from known isolates as the reference, 1349 HMP reads were identified as from either TR or VR from the human samples. By assembling predicted TR reads, we were able to identify additional TR variants (HMP1–5), but only two (HMP4 and HMP5) are complete. **Figure 2B** shows the neighbor-joining tree of these newly identified TR variants with the three TR types identified from sequenced isolates. Although more TR variants were identified from the HMP datasets, the tree of the TRs still shows two main branches—separating the SP32 from the ATCC35405-type—supporting the existence of two main types of DGR systems in *T. denticola*.

We added the predicted TRs from the HMP datasets to the reference TRs and searched for additional reads that contain either TR or VR in the HMP datasets. In total, we identified a total of 1,585 reads that can be recognized as either TR or VR. We used this collection of reads to study the distribution of DGR systems in the human population and the hypervariation patterns in VRs. DGR systems were identified in 80 human samples, and in total 633 reads—including all reads that matched with the three reference TRs and HMP1–HMP5—were recognized as TRs in those individuals.

We found that some individuals (or samples) had multiple types of TR, and almost half of the samples exhibited two or more types of TR. A few individuals had up to six types of TR. Despite the higher abundance of the ATCC35405-type TR in sequenced isolates (7 out of 9), the SP32-type TR is more abundant among the human population, being found in the most samples and accounting for the most reads (**Table 2**). ATCC35405-type and HMP1 TR are less abundant, but are still more prevalent than the other types of TRs.

**Table 2:** Population-wise analysis related to DGR system in human microbiome.

Reference TR	# of detected sample	Total reads
ATCC35405	25	78
SP37	12	23
**SP32**	**46**	**267**
HMP1	24	76
HMP2	10	40
HMP3	11	40
HMP4	10	36
HMP5	17	73


*The most prevalent TR (SP32) among the human microbiomes is shown in bold.*

Samples with detected TRs were only from oral sites (consistent with the distribution of *T. denticola*), and among oral sites was more common in subgingival plague and palatine tonsils (**Table 3**). *T. denticola* DGR systems were found proportionally significantly more in subgingival plaque (88% of the samples) than supragingival plague (29% of the samples); the difference would be even bigger if considering the difference of dataset sizes (on average, the supragingival plaque dataset is twice of that of the subgingival plaque dataset). Our previous study (Wu et al., 2012) had shown that *T. denticola* is found ubiquitously in supragingival plaque datasets (77% of the datasets), using the existence of the integron repeat sequences (*attC*) as tags. The ubiquitous existence of *T. denticola* in supragingival plaques is also supported by reads mapping: 75% of the supragingival samples each have at least 6,335 *T. denticola* reads [mapped to *T. denticola* ATCC35405 reference genome by bowtie2 (Langmead and Salzberg, 2012)]. However, *T. denticola* exists in lower abundance in supragingival plaques than in subgingival samples: the median number of *T. denticola* reads is 13,662 for supragingival plaque samples, whereas the median number is 51,645 for subgingival samples (see boxplots in **Supplementary Figure S5**). The abundance difference, therefore, contributed to the difference between the proportions of supragingival plaques and subgingival plaques with TRs detected. However, we noticed that some supragingival samples have many *T. denticola* reads, but lack detectable TR reads or have few TR reads. For example, a sample (ID: SRS011126) has 206,744 *T. denticola* reads, but has no TR reads. The two supragingival plaque samples that have the most *T. denticola* reads are SRS016331 (which has 645,178 *T. denticola* reads) and SRS023595 (which has 683,678 *T. denticola* reads) but each has only one TR read. These samples are likely to carry *T. denticola* isolates that lack DGR systems (however, the biological implications remain to be explored).

**Table 3:** Locations where TR and VR have been identified in HMP dataset.

Body site^∗^	# of detected sample	Total samples	Average dataset size (bp)	% of samples with TR detected
Buccal mucosa	10	122	156,880,336,215	8%
Palatine Tonsils	3	6	202,296,114,550	50%
Saliva	2	5	130,240,994,480	20%
Subgingival plaque	7	8	190,022,947,812	88%
Supragingival plaque	37	128	366,332,894,701	29%
Throat	1	7	180,480,064,986	14%
Tongue dorsum	20	136	487,411,177,528	15%


*^∗^Body sites (including stool, vagina) that have no samples with detected TRs are not shown in this table.*

### Hypervariation Introduced by the DGR Systems in *T. denticola*

**FIGURE 5:**
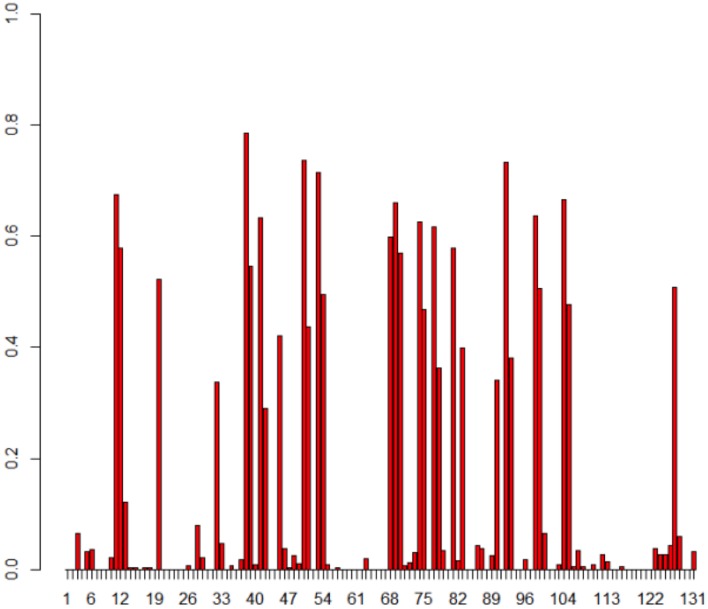
**Positional frequency of substitutions in the *T. denticola* DGR systems.** The *X*-axis shows each position in the TR and *Y*-axis is how often each position in TR is replaced in VRs. Each bar displays how frequently that position is substituted, and empty positions (no bars) represent either non-conserved positions between TRs, or positions with no observed substitutions in VRs. Note that we studied the TR-to-VR variation pattern using only positions that are conserved among TR sequences (so we won’t confuse variant TR sequences as mutations between the TR and VR sequences); a total of 335 TRs and 503 VRs were used for this analysis.

Since there are different TR variants within *T. denticola*, it is important to separate and exclude nucleotide variations found among TRs from the variation patterns generated in VRs. 335 reads of TR predicted to be closely related with complete TRs (three reference TRs, HMP4, and HMP5) were used for the identification of TR conservation. We first determined the conserved positions of the TR within TRs, and then used only those conserved regions to enumerate TR-to-VR nucleotide and amino acid variations. **Figure 5** illustrates the positional frequency of DGR substitutions (from TR to VR), revealing two distinctive frequency ranges: the first group of sites has very low variations (with a frequency between 0 and 0.1) and the second set of sites has a high frequency of variation (the hypervariation sites).

**FIGURE 6:**
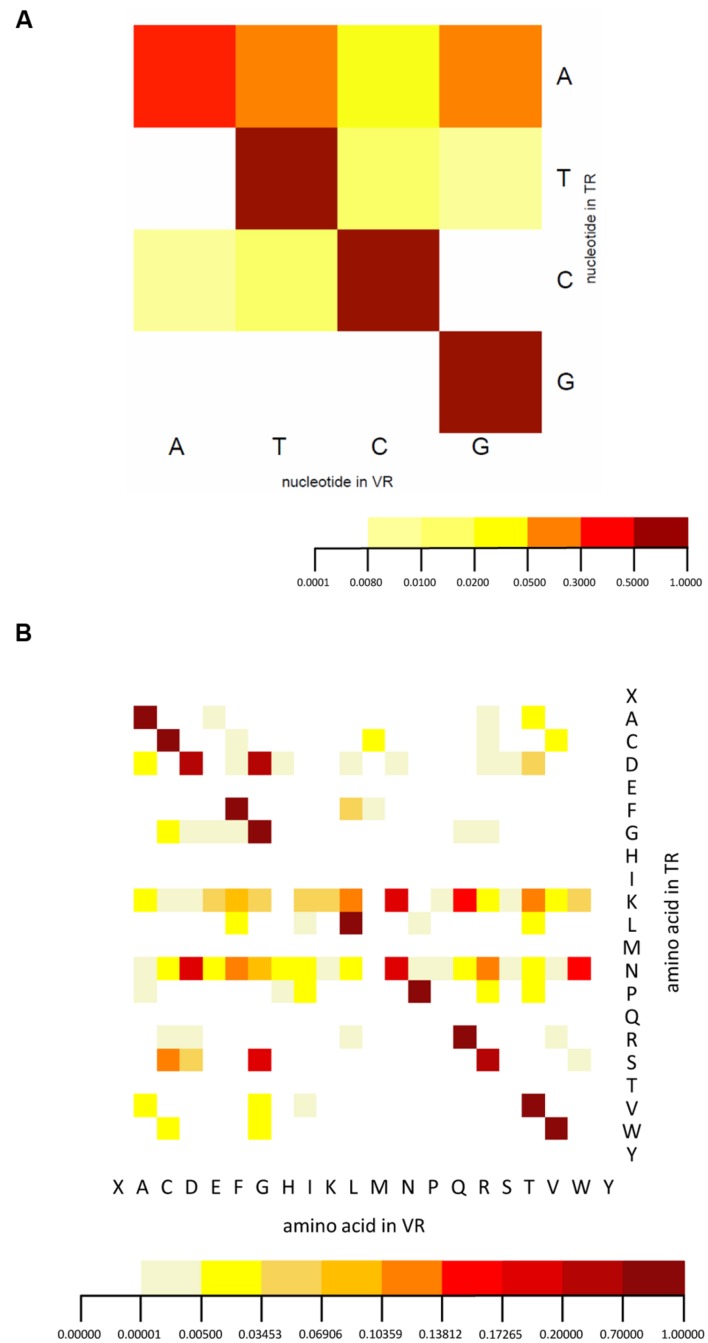
**Nucleotide and amino acid substitution bias of the DGR system.** **(A)** The nucleotide substitution frequency of the DGR system confirms the mutational bias toward adenine. **(B)** The amino acid mutation pattern suggests lysine and asparagine codons in the TR are the targets for hypervariation. Threonine and glutamine codons are not detected in either TR or VR sequences. Note “X” symbolizes stop codons. Dark red represents the highest frequency, orange medium frequency, and light yellow the lowest frequency. A total of 335 TRs and 503 VRs were used for this analysis.

We analyzed the spectrum of nucleotides and corresponding amino acid substitutions resulting from hypervariation. In nucleotide mutation, adenine nucleotides are most hyper-mutated, while rare mutations of other residues are also found (**Figure 6A**), consistent with the known adenine targeting of DGR RTs. The reason that we identified some rare mutations is probably a result of the large number of HMP samples. The amino acid variation we observed is very similar to that of human intestine viral DGRs (Minot et al., 2012) with some differences. The most significant difference is that hypervariation includes both lysine and asparagine codons for *T. denticola* associated DGR systems, while only asparagine codons were observed to be hypervariated in the human virome DGR systems, whose TRs lack lysine codons entirely (Minot et al., 2012) (**Figure 6B**) [but variations involving lysine codons are observed in other DGR systems (Doulatov et al., 2004; Schillinger et al., 2012)]. It has been shown that the asparagine-targeting hypervariation in viral DGR systems allows maximal chemical diversification of the encoded amino acids while avoiding the formation of stop codons; the same explanation probably does not apply to hypervariation targeting lysine codons.

**FIGURE 7:**
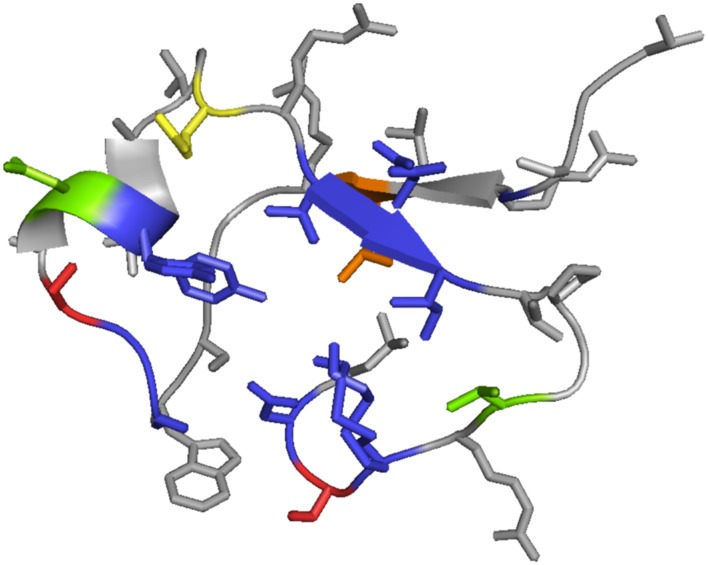
**Mapping of VRs onto the 3D structure of the C-lectin fold domain of TvpA (residue 286–321 are shown).** Different colors indicate different hypervariation targeting sites. Blue represents asparagines, yellow indicates lysines, green is either asparagine or lysine, red is either asparagine or aspartic acid, and orange represents a residue that varies among the reference TRs (it is isoleucine in Hmp5 and lysine in other reference TRs).

We mapped the identified variable residues onto the tertiary structure of C-type lectin domain of TvpA (see **Figure 7**) to visualize and locate the partiality in mutagenesis of DGR at the structural level. The mapping revealed that the VR was exposed on the outside of the protein, as previously reported. This might indicate that targeted gene products are primarily exposed to the extracellular environment and communicate with other living organisms. All side chains of hypervariable amino acids are highlighted on the outer surface of TvpA (**Figure 7**).

## Discussion

Few studies have focused on *T. denticola* isolates, let alone variation within their TRs (Doulatov et al., 2004; Schillinger and Zingler, 2012; Schillinger et al., 2012). Here we first identified new TR variants of *T. denticola* from sequenced isolates (total of three types), and more variants that could be extracted from in the HMP dataset. Interestingly, the three characterized TR variants indicate how similar these isolates are to one another, as suggested by the phylogenetic tree. We observed that the DGR locus in *T. denticola* is organized in a similar fashion in every isolate, and is of the Group 1a type (Schillinger et al., 2012). The DGR system is always located next to an accessory gene common to all the genomes, encoding an HRDC domain, which facilitates the denaturation of DNA strands, presumably allowing access to other proteins (Liu et al., 1999), and potentially collaborating with RT in reverse transcription of TR RNA to VR cDNA, or in the homing reaction. Despite the association of phages with the DGR systems of some organisms, we did not find any association between known bacteriophage genes and the DGR locus in *T. denticola*.

In the metagenomic database we found individuals with multiple TRs. The numerous TRs within an individual—added to the variation that arises from each, and the multiple target genes in some genomes—suggest that the potential advantage *T. denticola* derives from varying the cell surface receptors. However, the majority of individuals expressed only a single TR, probably reflecting the relatively rare colonization of an individual by multiple *T. denticola* clones. Despite our success determining TRs and VRs in the HMP dataset, we were not able to identify the associated RT genes because the length of current HMP reads is too short to confidently assign gene identities; also the *T. denticola* genome is at low abundance in these datasets, and cannot be assembled well. But the DGR RTs could be identified in the complete/draft *T. denticola* genomes, and this information allowed us to precisely locate the main DGR modules.

Many questions concerning DGR systems are still unanswered, and their complete mechanism is not yet understood. Here we analyzed the relatively unstudied DGR system of *T. denticola*, and discovered a DGR system that is dynamic and complex, as we report. And while we cannot identify complete *T. denticola* DGR loci in human microbiomes—because of the low abundance of *T. denticola* in healthy human populations—we found characteristics that are not observed in other organisms. We find that different *T. denticola* genomes have clearly different DGR types, suggesting multiple acquisitions and losses in this species, and that these and additional types are present in natural populations, sometimes in the same human host. Further, the DGR system of *T. denticola* has an expanded spectrum of hypervariation, compared to the *Bordetella* bacteriophage DGR (Le Coq and Ghosh, 2011). While currently impossible to know, it is easy to imagine that the *T. denticola* target *tvpA* gene takes part in biofilm formation or adhesion to host surfaces and see a ligand that is itself variable: the *Bordetella* bacteriophage Mtd protein binds a *Bordetella* surface protein that is a virulence factor, which in turn binds to human host cells.

## Conclusion

We have used genomic and metagenomic approaches to characterize the *T. denticola* DGR system. This allowed us to identify the DGR system in a subset of the *T. denticola* reference genomes—multiple TR variants that were not discovered previously—and how this DGR system is distributed in the human population. The metagenomic information was analyzed to reveal the substitution bias within the DGR system of *T. denticola*. Our analysis of *T. denticola* DGR systems using both individual genomes and the metagenomes (of a mixture of species) demonstrated the importance of combining individual genomes and metagenomes for studies of important genetic elements.

## Availability of Supporting Data

Sequences of putative TRs and VRs identified from the human microbiomes, information of identified target genes and accessory genes from isolates (including the coordinates and protein/nucleotide sequences) are available at our website http://omics.informatics.indiana.edu/mg/tDGR.

## Author Contributions

SN carried out the analysis and drafted the manuscript. HL participated in the analysis. TD participated in the analysis and helped to draft the manuscript. YY conceived of the study, and participated in its design and coordination and helped to draft the manuscript. All authors read and approved the final manuscript.

## Conflict of Interest Statement

The authors declare that the research was conducted in the absence of any commercial or financial relationships that could be construed as a potential conflict of interest.

## Footnotes

1 www.pymol.org

## Abbreviations

DGR: diversity generating retroelements
RT: reverse transcriptase
TR: template region
VR: variable region
