# Supplementary material for: Genomic and Metagenomic Analysis of Diversity-Generating Retroelements Associated with Treponema denticola
Source: Front Microbiol. 2016 Jun 3;7:852. doi: 10.3389/fmicb.2016.00852 (PMC4891356; doi:10.3389/fmicb.2016.00852)
Supplement: Supplementary file 1 [file Image_1.PDF]

```
TR        1  GTCAGGCTCTAACCGTGTTAAACGCGGCGGCAGCTGGAACAACA  44
TDE0572   1  GTCAGGCCCTTACCGCGTCAGGCGCGGCGGCAGTTGGGGCGGCA  44
TDE0945   1  GTCGGGTTCTTACCGCGTCCTACGCGGCGGCAGTTGGTACTTCT  44
TDE1056   1  ATCAGGCTCTTACCGTGTTCTACGCGGCGGCAGCTGGTACGATA  44
TDE2101   1  GTCGGGCTCTGCCCGTGTTGAACGCGGCGGCAGCTGGCTCAACT  44
TDE2239   1  GTCGGGGTCTGCCCGTGTTGATCGCGGCGGCAGCTGGGACAACG  44
TDE2269   1  GTCAGGCTCTGGCCGTGTTTTACGCGGCGGCAGCTGGGCCGGCA  44
TDE2515   1  GTCAGGCTCTAACCGTGTTTTACGCGGCGGCAGCTGGGACAACT  44

TR       45  ACGCGAACAACTGCACTGTAGGCAAACGGAATAACAACAGTCCT  88
TDE0572  45  GCGCGGGCGGCTGCACTGTAGGCAGACGGGAGGACTACAGTCCT  88
TDE0945  45  ACGCGCACTACTGCACTGTAGGCGTACGGGGCTACTACAGTCCT  88
TDE1056  45  GCGCGAAGAACTGCACTGTAGGCTTTCGGTTTAACCGCGACCCT  88
TDE2101  45  ACGCGTACGGCTGCACTGTAGGCGTACGGTACTGCGTCACTCCT  88
TDE2239  45  GCGTGAGGTACTGCAGTGTAGGCTTTCGGTTCAGCTGCAGTCCT  88
TDE2269  45  GCGCGGACTACTGCGCTGTAGGCGAACGGGTCAACATCAGTCCT  88
TDE2515  45  ACGCGGAGGGCTGCACTGTGGGCACCCGGGGTAACGGCAACCCT  88

TR       89  GACAACAGGAACAACAATCTTGGCTTCCGCTTGGCTTGTCGGC  131
TDE0572  89  GACAGCAGGTACAACTATCTTGGCTTCCGCCTGGCTTGGCGCC  131
TDE0945  89  GACTACTGGAGCTTCTATCTTGGCTTCCGCTTGGCTTGCCGGC  131
TDE1056  89  GGCTACGGGAACTTAGATATTGGCTTCCGCTTGGCTTGGCGGC  131
TDE2101  89  GGCAGCAGGAGCGACAATCTTGGCTTCCGCCTGGCTTGTCGGC  131
TDE2239  89  GGCCACAGGTACTACGGTCTTGGCTTCCGCCTGGCTTGTCTGC  131
TDE2269  89  GGCGTCAGGTGCAGCGATCTTGGCTTCCGCCTGGCTTGCCGGC  131
TDE2515  89  GGCAACAGGGGCAACCTTCTTGGCTTCCGCTTGGCTTGCCGGC  131
```

Supplementary Figure 1. Multiple alignment between the TR and VR regions (from six target genes) found in ATCC35405.
