# Supplementary material for: Genomic and Metagenomic Analysis of Diversity-Generating Retroelements Associated with Treponema denticola
Source: Front Microbiol. 2016 Jun 3;7:852. doi: 10.3389/fmicb.2016.00852 (PMC4891356; doi:10.3389/fmicb.2016.00852)
Supplement: Supplementary file 2 [file Image_2.PDF]

ATCC35405: three target proteins predicted to be lipoproteins

MKTSNAKTKAYAFRGGSALITAAVLALVALFG**MTGC**PNNAGGGGSGSGNSG	TDE0572
MKKFFVLFLAILFVS**VSAC**KNPFFKNMLDKDSGS	TDE2101
MKNTNSKLKTKVLNRAISITALLLAAGVL**LTGC**PTGQGKSGGGESSEV	TDE2269

SP32: seven target proteins predicted to be lipoproteins

MKKIFVLFLAILFVS**VSAC**KNPFFKNMLDKDSG	HMPREF9731_00920
MKKIFVLFLAILFVS**VSAC**KNPFFKNMLDKDSG	HMPREF9731_01043
MKNTNSKLKTKVLNRAVSITALLLAAGVL**LTGC**PTGQGKSGGGESSE	HMPREF9731_01071
MTKFKTKHKAHALRKTGAAVLITAALLAVGLL**FTSC**KQASGSNSSDSDGS	HMPREF9731_02040
MKKFNSNKNKKAKAFTVKGAAALIIAAILAAALL**FTGC**NQPSGSSSGGNSGN	HMPREF9731_02191
MTKFKTNNKKARAFTVKGAAALIIAAILAAALL**FTGC**NQPSGSSSGGNSGG	HMPREF9731_02488
MKHKVKTILFAGLALIALFG**MTAC**PNNAGGGGVGSFED	HMPREF9731_02587

Supplementary Figure 2. Selected examples of lipoprotein signal peptide (SpII) predictions in SP23 and ATCC35405. Only the N-terminal sequences of the target proteins are shown here for clarity. The predicted lipoboxes are highlighted in red.
