# Supplementary material for: Genomic and Metagenomic Analysis of Diversity-Generating Retroelements Associated with Treponema denticola
Source: Front Microbiol. 2016 Jun 3;7:852. doi: 10.3389/fmicb.2016.00852 (PMC4891356; doi:10.3389/fmicb.2016.00852)
Supplement: Supplementary file 3 [file Image_3.PDF]

ATCC35405

TR CCGCGTC**A**GGCTCT**AA**CCGTGTT**AAA**CGCGGCGGC**A**GCTGG**AA**C**AA**C**AA**CGCG**AA**C**AA**C
VR CCGCGTCAGGCTCT**GG**CCGTGTT**TT**ACGCGGCGGCAGCTGG**GC**C**GG**CA**G**CGCG**G**AC**T**AC

TR TGC**A**CTGTAGGC**AAA**CGG**AA**T**AA**C**AA**C**A**GTCCTG**A**C**AA**C**A**GG**AA**CA**A**C**A**ATCTTGGCTT
VR TGC**G**CTGTAGGC**G**AACGG**GT**CAACA**T**CAGTCCTG**G**C**GT**C**A**GG**TG**CA**G**C**G**ATCTTGGCTT

TR CCGC**T**TGGCTTG**T**CGGCC**C**
VR CCGC**C**TGGCTTG**C**CGGCC**T**taa

SP32

TR GCGCGGCTGGCGTT**AA**CCGCGTC**AAA**CGCGGCGGT**A**GCTGG**AA**C**AA**T**AA**CGCG**AA**G**AA**C
VR GCGCGGCTGGCGTT**GG**CCGCGTC**GG**ACGCTGCGGT**G**GCTGG**T**AC**G**AT**G**ACGCG**G**AG**CT**C

TR TGCGTCGTCGGT**AAA**CGG**AA**C**AA**C**AA**C**AA**CCCCGGC**AA**C**A**GC**AA**CG**A**C**A**ATCTTGGCTT
VR TGCGTCGTCGGT**TCG**CGG**GT**CAAC**TG**C**T**ACCCCGGC**G**ACAGC**G**ACGAC**G**ATGTTGGCTT

TR TCG**TG**TGGCTTGTCGCCCC
VR TCG**GC**TGGCTTGTCGCCCCtga

Supplementary Figure 3. Alignment of putative TR and VR regions found in ATCC35405 and the SP32 isolates. VR regions of the target genes in the core DGR systems are used for the alignments (there are multiple target genes found in these isolates). Stop codons in the VR regions are shown in lower case. Adenines in TR sequences are highlighted in bold, and the corresponding bases in VR are highlighted in bold, if they are different from the bases in TR. Mismatches at the 3'-end are potential elements that might be important for homing (similar to the IMH in *Bordetella* phage), and are highlighted in red.
