# Supplementary material for: Genomic and Metagenomic Analysis of Diversity-Generating Retroelements Associated with Treponema denticola
Source: Front Microbiol. 2016 Jun 3;7:852. doi: 10.3389/fmicb.2016.00852 (PMC4891356; doi:10.3389/fmicb.2016.00852)
Supplement: Supplementary file 4 [file Image_4.PDF]

```
atcc35405 CCGCGTCAGGCTCTAACCGTGTTAAACGCGGCGGCAGCTGGAACAACAAC
sp37      CCGCGTCAGGCTCTAACCGTGTTAAACGCGGCGGCAGCTGGAACAACAAC
sp32      GCGCGGCTGGCGTTAACCGCGTCAAACGCGGCGGTAGCTGGAACAATAAC

atcc35405 GCGAACAACTGCACTGTAGGCAAACGGAATAACAACAGTCCTGACAACAG
sp37      GCGAACAACTGCACTGTAGGCAAACGGAACAACAACAGTCCTGACAACAG
sp32      GCGAAGAACTGCGTCGTCGGTAAACGGAACAACAACAACCCCGGCAACAG

atcc35405 GAACAACAATCTTGGCTTCCGCTTGGCTTGTCGGCCC
sp37      GAACAACAATCTTGGCTTCCGCTTGGCTTGTCGGCCC
sp32      CAACGACAATCTTGGCTTTCGTGTGGCTTGTCGCCCC
```

Supplementary Figure 4. Alignment of the full-length TR sequences identified from three *T. denticola* isolates. The positions with different bases in sp32 and atcc35405 are highlighted in red, and the only position that differs between atcc35405 and sp37 is highlighted in blue.
