# Supplementary material for: Genomic and Metagenomic Analysis of Diversity-Generating Retroelements Associated with Treponema denticola
Source: Front Microbiol. 2016 Jun 3;7:852. doi: 10.3389/fmicb.2016.00852 (PMC4891356; doi:10.3389/fmicb.2016.00852)
Supplement: Supplementary file 5 [file Image_5.PDF]

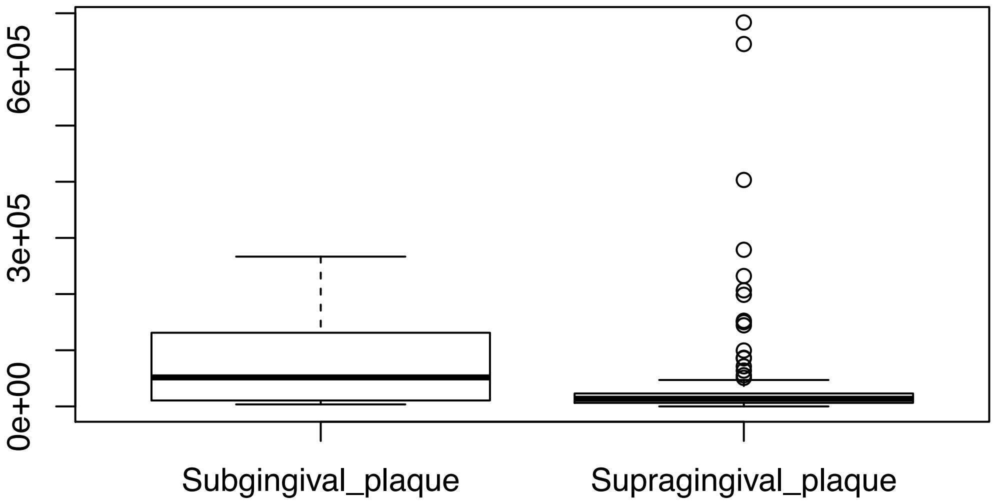

Supplementary Figure 5. Boxplots for the number of *T. denticola* reads identified from supragingival plaque and subgingival plaque samples.
